# Follow up duration of phase III Multiple Myeloma Clinical Trials: A systematic review

**DOI:** 10.1002/jha2.680

**Published:** 2023-06-29

**Authors:** Mohammad O. Ali, Hafez M. Abdullah, Khaldun Obeidat, Rajshekhar Chakraborty, Samer Al Hadidi

**Affiliations:** ^1^ Department of Internal Medicine Sanford School of Medicine University of South Dakota Sioux Falls South Dakota USA; ^2^ Department of Internal Medicine John H Stroger Jr Hospital of Cook County Chicago Illinois USA; ^3^ Multiple Myeloma and Amyloidosis Program Herbert Irving Comprehensive Cancer Center, Columbia University Irving Medical Center New York New York USA; ^4^ Myeloma Center, Winthrop P. Rockefeller Cancer Institute University of Arkansas for Medical Sciences Little Rock Arkansas USA

**Keywords:** clinical trials, follow‐up duration, long‐term follow‐up, multiple myeloma

## Abstract

Long‐term follow‐up of multiple myeloma (MM) clinical trials are needed to assess long‐term outcomes. We aimed to investigate the length of follow‐up of all phase III MM clinical trials. Median follow‐up duration of clinical trials of newly diagnosed MM was longer when compared to relapsed/refractory MM clinical trials (42.7 vs. 20.5 months, respectively). The follow‐up duration of phase III clinical trials in MM is relatively short when compared to the improved outcomes in the current era. Efforts should be made to facilitate long‐term clinical trials follow‐up and/or publication of results of updated results.

1

Multiple myeloma (MM) is a plasma cell malignancy where cure is achievable in some patients with the use of novel therapies and high‐dose chemotherapy followed by autologous stem cell transplantation (ASCT) [1]. Outcomes of patients with MM continue to improve especially for patients with standard‐risk disease. The 5‐year relative survival rates between 2011 and 2017 of patients with MM were 56% based on the Surveillance, Epidemiology, and End Results (SEER) database [2]. In an updated follow‐up of SWOG S0777 study with median follow‐up of 84 months, the median overall survival (OS) of bortezomib, lenalidomide, and dexamethasone (VRd) was not reached, and the median progression free survival (PFS) was 41 months [3]. Real‐world data showed 10‐year OS rates of 29% and 58% for high‐risk and standard‐risk disease, respectively. Patients were treated with VRd, and upfront ASCT was done in 75% of patients [4]. Given the improvement in outcomes of patients with MM, long‐term follow‐up of phase III clinical trials is crucial. Our study aimed to investigate the length of follow‐up of all phase III MM clinical trials.

We searched www.clinicaltrials.gov for all phase III completed clinical trials that studied MM. Search terms included *myeloma, multiple myeloma, plasma cell dyscrasia*. We limited our studies to phase III studies reported as completed. Data cut‐off data of September, 2022. Systematic review was conducted using study identifier/s, principal investigator name, and/or study title using PubMed, Cochrane, and Google Scholar search engines to identify the most recent publication to be included in the analysis. Given the publicly available data, this study was considered exempt from the University of Arkansas for Medical Sciences.

Data on clinical trials characteristics were collected, including planned median follow‐up duration reported at www.clinicaltrials.gov and the final reported actual median follow‐up duration for survival outcomes and/or response‐related outcomes retrieved from the latest publication. We collected data on estimated original enrollment, actual number of patients enrolled, study start date and completion date, date of last published update, study location/s, disease status (newly diagnosed vs. relapsed/refractory), race/ethnicity of enrolled patients, study primary outcome/s, and reported follow‐up durations. All statistical analyzes were done using R statistical software version 4.0.5 (R Project for Statistical Computing) [5].

A total of 169 studies were retrieved (Table S1). Out of these 45 studies were excluded due to irrelevance, duplication, and/or early termination. A total of 124 clinical trials were included in our final analysis. The average number of patients enrolled in completed phase III MM clinical trials was 371 (range: 4–1913). Older adults aged ≥65 years old were included in 103 (83.1%) clinical trials. More studies targeted newly diagnosed MM (NDMM) versus relapsed/refractory (R/R) MM (63.7% vs. 33.1%, respectively). Survival‐related endpoints were reported in 63 (50.8%) clinical trials compared to response‐related endpoints in 30 (24.2%) clinical trials. Other primary outcomes included safety‐related outcomes in 8 studies (6.5%), other outcomes in 15 studies (12.1%), and primary outcomes were not provided in 8 studies (6.5%) (Table 1).

**TABLE 1:** Characteristics of included phase III Multiple Myeloma Clinical Trials

Characteristics	No. of Trial (%) (*n* = 124)
**Target population**	
Included age = ≥65 years old	103 (83.1%)
Newly diagnosed multiple myeloma	79 (63.7%)
Relapsed/refractory multiple myeloma	41 (33.1%)
Other	3 (2.4%)
Not reported	1 (0.8%)
**Geographical distribution**	
US only	28 (24.6%)
US and a global site	29 (25.4%)
Global sites only	57 (50%)
**Primary outcome reported**	
Survival‐related outcome	63 (50.8%)
Response‐related outcome	30 (24.2%)

Of survival‐related outcomes (*n* = 63), the most common primary outcome was either PFS, time to progression or event free survival in 54 studies (85.7%), whereas OS was the primary endpoint in 9 studies (14.3%). Race and/or ethnicity of participating patients were reported only in 27 (21.8%) clinical trials. A total of 94 studies (75.8%) had subsequent publications with the duration of follow‐up reported in only 56 publications. The rest of clinical trials (*n* = 30) only reported intended follow‐up duration at www.clinicaltrials.gov with no subsequent publication/presentation.

The planned median follow‐up durations from www.clinicaltrials.gov were 42.8 (range: 10.9–132), 36 (range: 10.9–132), 24 (range: 6–70) months for OS, PFS, and duration of response (DOR), respectively. The most recent median follow‐up durations retrieved from the latest publication were 44.3 (range: 27.8–76), 44 (range: 15.9–120), and 28 (range: 10.4–77.2) months for OS, PFS, and DOR, respectively. The actual median follow‐up duration for all outcomes from latest publication is 35.2 months (range: 10.4–120 months). Median follow‐up duration of NDMM clinical trials was longer when compared to R/R MM clinical trials (42.7 [range: 12–120] vs. 20.5 [range: 10.4–48.6] months, respectively) (Figure 1).

**FIGURE 1:**
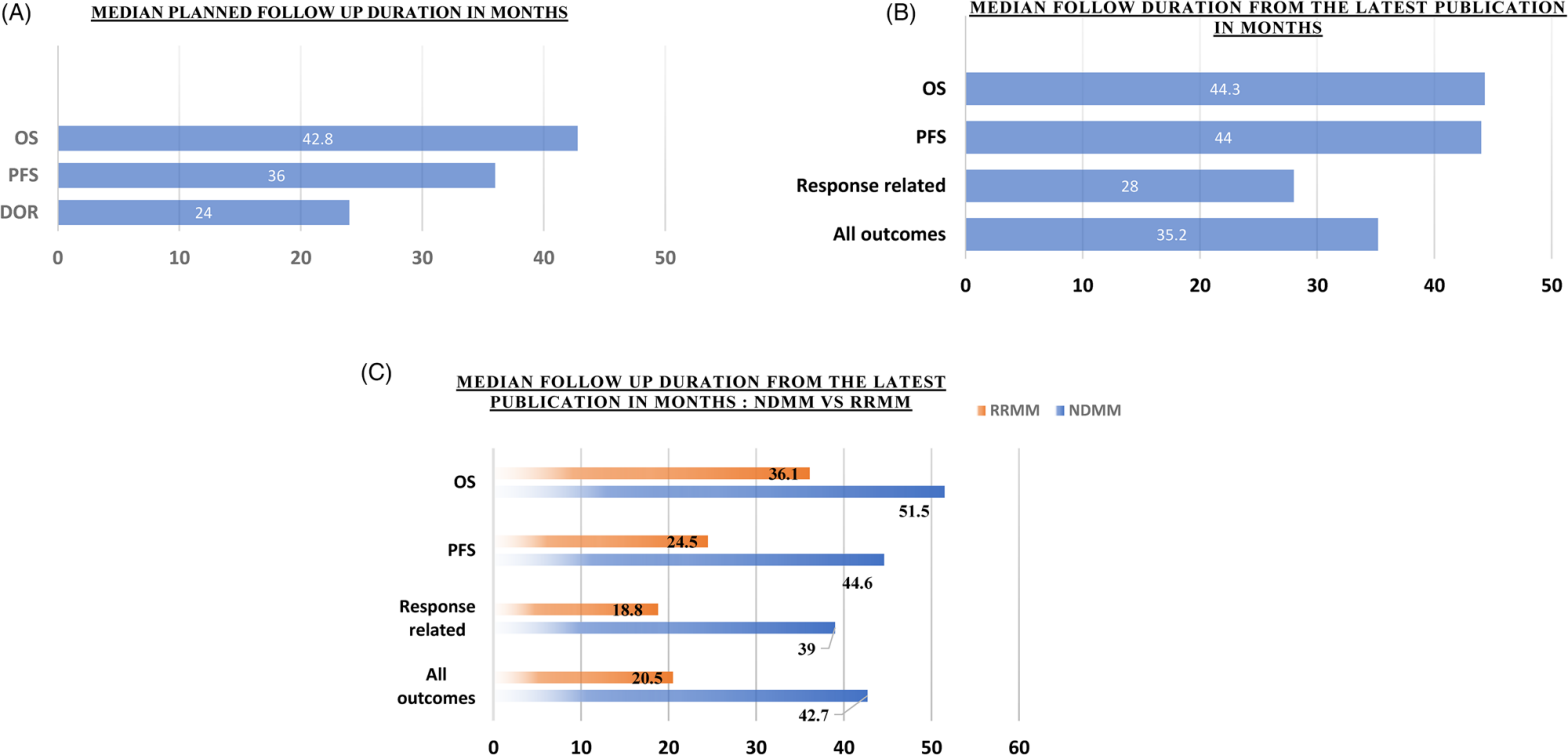
Median planned and actual follow‐up duration of Multiple Myeloma Clinical Trials: Part (A) shows the median planned follow‐up duration for overall survival (OS), progression free survival (PFS), and duration of response (DOR); part (B) shows the actual median follow‐up duration for OS, PFS, response‐related outcomes, and all outcomes; part (C) compares the actual median follow‐up duration for OS, PFS, response‐related outcomes, and all outcomes between newly diagnosed multiple myeloma (NDMM) and relapsed refractory multiple myeloma (RRMM) patients.

Our analysis showed that median follow‐up duration of phase III completed clinical trials in MM is relatively short when compared to the actual survival of MM patients. For NDMM, the median follow‐up duration was approximately 3.5 years. In patients with NDMM, survival outcomes continue to improve [1, 3, 5, 6]. Longer follow‐up duration of trial participants after the end of the scheduled trial follow‐up period can provide important information on both safety and efficacy outcomes. With the improvement of outcomes for patients with NDMM and RRMM, we believe that longer follow‐up duration for phase III clinical trials is needed. Short follow‐up duration in phase III clinical trials may underestimate potential benefits of treatments investigated and fail to detect safety issues, which can take much longer to emerge. This is becoming more relevant in MM as outcomes continue to improve.

Randomized clinical trials can be very expensive. The cost of longer follow‐up duration may add to the overall cost of conducting clinical trials especially with the need of logistical considerations. The value of long‐term follow‐up of clinical trials in the field of oncology is highly significant. Multiple large clinical trials reported long‐term follow‐up durations which make this feasible [1, 7]. Clinical trial extension by record linkage may be a potential solution though it remains underused [8, 9]. Results after long‐term follow‐up may lead to different interpretations that were different from those of the original articles [10, 11, 12]. In NDMM, longer follow‐up showed improved OS in patients who received ASCT which was not shown in initial publication [11]. In R/R MM, longer follow‐up showed improved OS with the use of daratumumab‐based regimen [12].

Our analysis showed that a significant number of subsequent publications for phase III clinical trials in MM did not report a follow‐up duration. Moreover, about one fourth of included clinical trials did not have subsequent publications/presentation. Future efforts should be made to facilitate publications of all phase III clinical trials regardless of the results and to ensure the reporting of follow‐up durations in updated publications.

Our analysis has few limitations. We did not account for any plans to report on longer follow‐up duration. Most of the actual publications reported longer follow‐up durations in most recent publications when compared to planned follow‐up provided in clinicaltrials.gov. We only included phase III clinical trials in our analysis. Despite those limitations, we believe that our analysis provides important information on the current follow‐up duration for all completed phase III clinical trials in the field of MM.

In conclusion, we found that the follow‐up duration of phase III clinical trials in MM is relatively short when compared to the improved outcomes in the current era. Efforts should be made to facilitate long‐term clinical trials follow‐up and/or publication of results of updated results.

## AUTHOR CONTRIBUTIONS

Samer Al Hadidi conceived the research idea. Mohammad O. Ali, Khaldun Obeidat, and Hafez M. Abdullah worked on data collection and study selection. Mohammad O. Ali and Samer Al Hadidi worked on review of literature, statistical analysis, and manuscript drafting. All authors wrote the initial draft of the manuscript. Rajshekhar Chakraborty and Samer Al Hadidi critically reviewed and approved the final submission of the manuscript.

## CONFLICT OF INTEREST STATEMENT

Authors declare that they have no conflict of interests.

## DATA SHARING/AVAILABILITY STATEMENT

Data request can be made to the corresponding author.

## Supporting information

Supporting Information
