# Supplementary material for: Follow up duration of phase III Multiple Myeloma Clinical Trials: A systematic review
Source: EJHaem. 2023 Jun 29;4(3):719–22. doi: 10.1002/jha2.680 (PMC10435726; doi:10.1002/jha2.680)
Supplement: Supplementary file 1 — Supporting Information [file JHA2-4-719-s001.docx]

**Supplementary material**

**Supplementary Table 1.** Included National Clinical Trial (NCT) studies

| **No.** | **NCT number** |  |
| --- | --- | --- |
| 1 | NCT01568866 |  |
| 2 | NCT01971658 |  |
| 3 | NCT01734928 |  |
| 4 | NCT00560053 |  |
| 5 | NCT00038233 |  |
| 6 | NCT00452569 |  |
| 7 | NCT01080391 |  |
| 8 | NCT01801436 |  |
| 9 | NCT00622336 |  |
| 10 | NCT01712789 |  |
| 11 | NCT00891384 |  |
| 12 | NCT00048230 |  |
| 13 | NCT00057564 |  |
| 14 | NCT01296503 |  |
| 15 | NCT01070862 |  |
| 16 | NCT01908621 |  |
| 17 | NCT00075829 |  |
| 18 | NCT00218855 |  |
| 19 | NCT00111319 |  |
| 20 | NCT02726581 |  |
| 21 | NCT00378222 |  |
| 22 | NCT02811978 |  |
| 23 | NCT00367185 |  |
| 24 | NCT00424047 |  |
| 25 | NCT01324947 |  |
| 26 | NCT01109004 |  |
| 27 | NCT01311687 |  |
| 28 | NCT00813150 |  |
| 29 | NCT00416273 |  |
| 30 | NCT02412878 |  |
| 31 | NCT02197221 |  |
| 32 | NCT00689936 |  |
| 33 | NCT00063726 |  |
| 34 | NCT00934154 |  |
| 35 | NCT00416208 |  |
| 36 | NCT01023308 |  |
| 37 | NCT01818752 |  |
| 38 | NCT01302392 |  |
| 39 | NCT00056160 |  |
| 40 | NCT01146834 |  |
| 41 | NCT00480363 |  |
| 42 | NCT01530594 |  |
| 43 | NCT01621672 |  |
| 44 | NCT01335399 |  |
| 45 | NCT03158688 |  |
| 46 | NCT00097981 |  |
| 47 | NCT00928486 |  |
| 48 | NCT01063179 |  |
| 49 | NCT00002548 |  |
| 50 | NCT00179647 |  |
| 51 | NCT00722566 |  |
| 52 | NCT00103506 |  |
| 53 | NCT00432458 |  |
| 54 | NCT00773747 |  |
| 55 | NCT00376883 |  |
| 56 | NCT00083876 |  |
| 57 | NCT01309334 |  |
| 58 | [NCT00732641](https://clinicaltrials.gov/show/NCT00732641) |  |
| 59 | NCT00443235 |  |
| 60 | NCT00420849 |  |
| 61 | NCT00083551 |  |
| 62 | [NCT00006232](https://clinicaltrials.gov/show/NCT00006232) |  |
| 63 | NCT00002878 |  |
| 64 | NCT00103662 |  |
| 65 | [NCT01564537](https://clinicaltrials.gov/show/NCT01564537) |  |
| 66 | NCT00546780 |  |
| 67 | NCT00064038 |  |
| 68 | [NCT03029234](https://clinicaltrials.gov/show/NCT03029234) |  |
| 69 | NCT02406144 |  |
| 70 | NCT00049673 |  |
| 71 | NCT00033332 |  |
| 72 | NCT00478777 |  |
| 73 | [NCT00008229](https://clinicaltrials.gov/show/NCT00008229) |  |
| 74 | NCT00405756 |  |
| 75 | NCT00017602 |  |
| 76 | NCT01102426 |  |
| 77 | NCT00434161 |  |
| 78 | NCT00004165 |  |
| 79 | NCT00602511 |  |
| 80 | NCT03402295 |  |
| 81 | NCT02495922 |  |
| 82 | NCT00232934 |  |
| 83 | NCT00002678 |  |
| 84 | NCT00417911 |  |
| 85 | NCT01413178 |  |
| 86 | NCT01239797 |  |
| 87 | NCT01916252 |  |
| 88 | NCT01160380 |  |
| 89 | NCT00633542 |  |
| 90 | NCT00217438 |  |
| 91 | NCT00270101 |  |
| 92 | NCT00002850 |  |
| 93 | NCT01910987 |  |
| 94 | NCT00200681 |  |
| 95 | NCT02112175 |  |
| 96 | NCT00002556 |  |
| 97 | NCT01539083 |  |
| 98 | NCT00950768 |  |
| 99 | NCT00416897 |  |
| 100 | NCT02288741 |  |
| 101 | NCT00205764 |  |
| 102 | NCT00083915 |  |
| 103 | NCT00222053 |  |
| 104 | NCT00507416 |  |
| 105 | NCT00344422 |  |
| 106 | NCT01191060 |  |
| 107 | NCT00111748 |  |
| 108 | NCT00001561 |  |
| 109 | NCT02322320 |  |
| 110 | NCT00093028 |  |
| 111 | NCT00657488 |  |
| 112 | NCT02419118 |  |
| 113 | NCT00177047 |  |
| 114 | NCT00430365 |  |
| 115 | NCT03357952 |  |
| 116 | NCT00038090 |  |
| 117 | NCT00514371 |  |
| 118 | NCT00090493 |  |
| 119 | NCT01345019 |  |
| 120 | NCT00205751 |  |
| 121 | NCT00861250 |  |
| 122 | NCT01208818 |  |
| 123 | NCT00838357 |  |
| 124 | NCT00207805 |  |
